# Effects of quality improvement in health facilities and community mobilization through women’s groups on maternal, neonatal and perinatal mortality in three districts of Malawi: MaiKhanda, a cluster randomized controlled effectiveness trial

**DOI:** 10.1093/inthealth/iht011

**Published:** 2013-06-26

**Authors:** Tim Colbourn, Bejoy Nambiar, Austin Bondo, Charles Makwenda, Eric Tsetekani, Agnes Makonda-Ridley, Martin Msukwa, Pierre Barker, Uma Kotagal, Cassie Williams, Ros Davies, Dale Webb, Dorothy Flatman, Sonia Lewycka, Mikey Rosato, Fannie Kachale, Charles Mwansambo, Anthony Costello

**Affiliations:** a UCL Institute for Global Health, 30 Guilford Street, London WC1N 1EH, UK; b MaiKhanda Trust, Area 14, Plot 56, Private Bag B437, Lilongwe, Malawi; c Institute for Healthcare Improvement, 20 University Road, 7th Floor, Cambridge, MA 02138, USA; d Cincinnati Children’s Hospital Medical Center, 3333 Burnet Avenue, Cincinnati, OH 45229, USA; e Women and Children First (UK), United House, North Road, London N7 9DP, UK; f The Health Foundation, 90 Long Acre, London WC2E 9RA, UK; g Government of the Republic of Malawi, Ministry of Health Reproductive Health Unit, off Paul Kagame Road, Lilongwe, Malawi; h Government of the Republic of Malawi, Ministry of Health, Capital Hill, Lilongwe, Malawi

**Keywords:** Quality improvement, Women’s groups, Maternal mortality, Neonatal mortality, Perinatal mortality, Malawi

## Abstract

**Background:**

Maternal, perinatal and neonatal mortality remains high in low-income countries. We evaluated community and facility-based interventions to reduce deaths in three districts of Malawi.

**Methods:**

We evaluated a rural participatory women’s group community intervention (CI) and a quality improvement intervention at health centres (FI) via a two-by-two factorial cluster randomized controlled trial. Consenting pregnant women were followed-up to 2 months after birth using key informants. Primary outcomes were maternal, perinatal and neonatal mortality. Clusters were health centre catchment areas assigned using stratified computer-generated randomization. Following exclusions, including non-birthing facilities, 61 clusters were analysed: control (17 clusters, 4912 births), FI (15, 5335), CI (15, 5080) and FI + CI (14, 5249). This trial was registered as International Standard Randomised Controlled Trial [ISRCTN18073903]. Outcomes for 14 576 and 20 576 births were recorded during baseline (June 2007–September 2008) and intervention (October 2008–December 2010) periods.

**Results:**

For control, FI, CI and FI + CI clusters neonatal mortality rates were 34.0, 28.3, 29.9 and 27.0 neonatal deaths per 1000 live births and perinatal mortality rates were 56.2, 55.1, 48.0 and 48.4 per 1000 births, during the intervention period. Adjusting for clustering and stratification, the neonatal mortality rate was 22% lower in FI + CI than control clusters (OR = 0.78, 95% CI 0.60–1.01), and the perinatal mortality rate was 16% lower in CI clusters (OR = 0.84, 95% CI 0.72–0.97). We did not observe any intervention effects on maternal mortality.

**Conclusions:**

Despite implementation problems, a combined community and facility approach using participatory women’s groups and quality improvement at health centres reduced newborn mortality in rural Malawi.

## Introduction

Although recent trends show a decline in maternal mortality from 984 per 100 000 live births during 2000–2004 (just before this trial) to 675 per 100 000 live births during 2006–2010,^1^,^2^ Malawi is off-track to meet Millennium Development Goal 5 (a three-quarters reduction in maternal mortality between 1990 and 2015).^3^ Neonatal mortality, at 31 per 1000 live births in 2006–2010,^2^ and perinatal mortality, at 40 per 1000 live births in 2006–2010,^2^ are also high and lag behind decreases in the number of child deaths but Malawi is on-track to meet Millennium Development Goal 4.^3^,^4^

The main direct causes of maternal death in Malawi are haemorrhage, sepsis, ruptured uterus and eclampsia; and the main indirect causes are HIV, malaria and anaemia,^5^ with underlying social causes including poverty, illiteracy and lack of knowledge. The main causes of neonatal death are prematurity, asphyxia and sepsis.^4^

To reduce deaths, supply side (health system) and demand side (community and individual) barriers to adequate antenatal, intrapartum and postnatal care must be addressed.^6^ Based on the three delays model (delays in deciding to seek care, reaching the place of care and receiving adequate care once there),^7^ an international consortium of partner organizations designed supply side (health facility quality improvement; FI) and demand side (community women’s groups; CI) interventions to reduce maternal and newborn mortality.^8^ The impact of both interventions on deaths was evaluated by a two-by-two factorial cluster randomized controlled trial (RCT). Separate process and economic evaluation studies were also evaluated and are reported elsewhere.^9^

Previous research has shown that community mobilization through women’s groups reduced neonatal mortality in India^10^ and Nepal.^11^ However, a similar RCT in Bangladesh found no impact probably, due to low population coverage of the intervention.^12^

Robust evidence of the effect of quality improvement interventions on population mortality rates in the developing world is lacking.^13^,^14^ We hypothesized that women’s groups could reduce maternal, perinatal and neonatal mortality rates by 30% through changes in care practices and healthseeking behaviour; and that quality improvement at health centres and hospitals, given higher rates of institutional delivery in Malawi, could have a similar effect through better antenatal, delivery and postpartum care.

## Methods

The design of the trial is reported in full elsewhere.^9^ The trial was registered as an International Standard Randomised Controlled Trial [ISRCTN18073903]. A locally registered non-government organization (NGO), MaiKhanda (Chichewa for ‘mother and newborn infant’) was set up in Malawi to implement the interventions.

### Study population: clusters and participants

**Figure 1:**
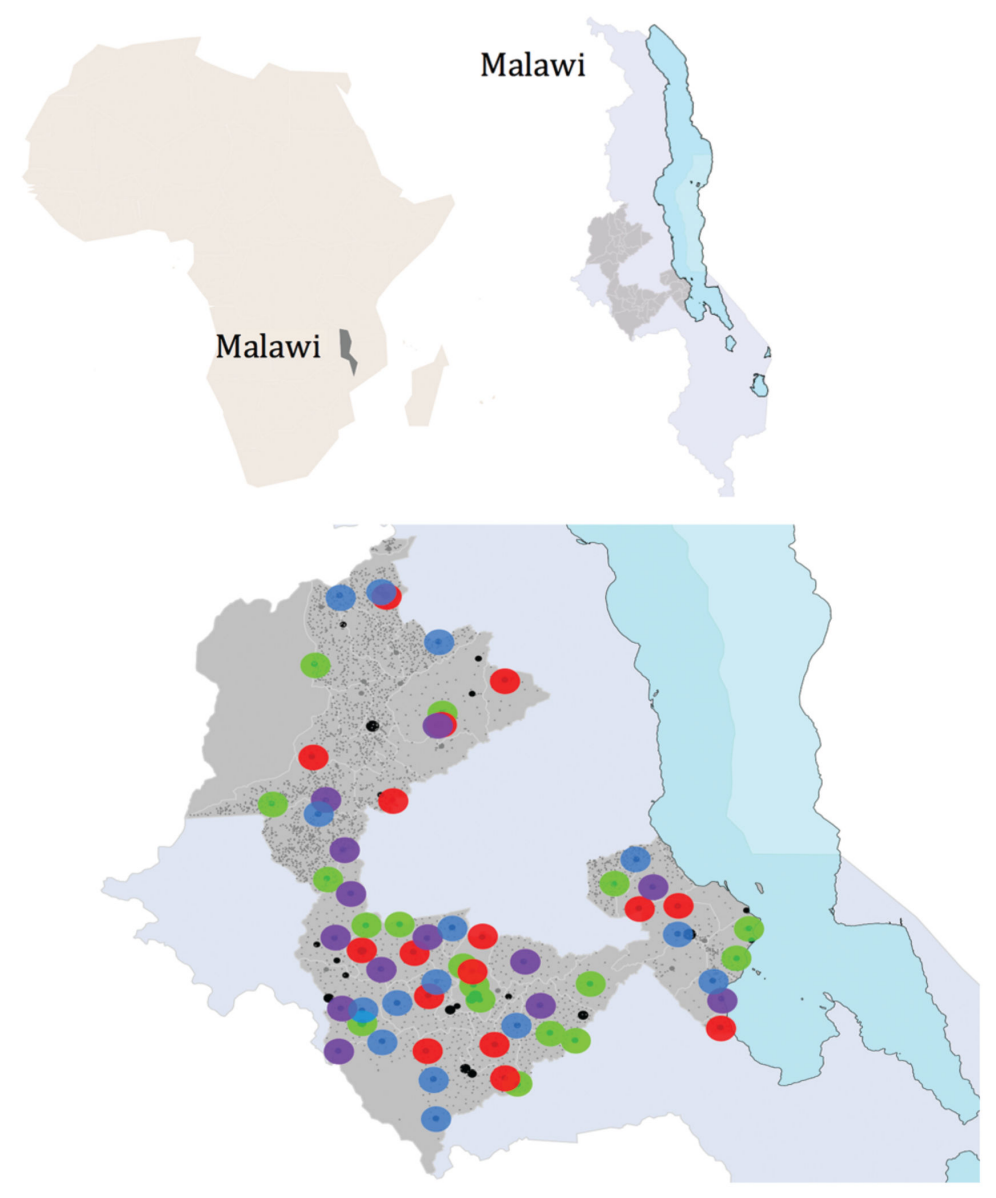
Map showing the central region of Malawi with the three districts where the randomized controlled trial took place shown in darker grey: Kasungu (top), Lilongwe (bottom) and Salima (right, by Lake Malawi). Key to randomized intervention areas: green: control clusters; red: quality improvement at health centres (MaiKhanda facilities intervention); blue: community women’s groups (MaiKhanda community intervention); purple: facility and community interventions together.

The study was conducted in Kasungu, Lilongwe and Salima, three districts of the central region of Malawi (Figure 1), chosen in a consultative process at a meeting of key stakeholders (from the district councils and health offices, Ministry of Health and local academic and NGOs) in October 2005. In 2010 in Kasungu, Lilongwe and Salima respectively, access to electricity was only 5.0%, 11.9% and 8.1%, only 3.7%, 14.6% and 8.8% of households had improved sanitation facilities, and female literacy rates were 72.3%, 66.7% and 56.9%.^2^ In 2011 Malawi had a human development index of 0.400 and an inequality-adjusted human development index of 0.272.^15^

Given that community mobilization takes place at village level and the quality improvement at health centres and hospitals, the unit of randomization was required to be a cluster of people. A suitable cluster for both interventions was the catchment population of a health centre. All health facilities in the three districts were included as clusters for the quality improvement trial except: (i) any facility providing comprehensive emergency obstetric care (CEmOC) functions (Caesarian section and blood transfusion) plus basic emergency obstetric care (BEmOC) signal functions (manual removal of placenta, manual vacuum aspiration, vacuum extraction, breech deliveries, parenteral antibiotics, magnesium sulphate, oxyticic drugs [ergonetrine or oxytocin]); (ii) any facility not offering any BEmOC functions (i.e. dispensaries); and non-functional facilities. We did not include CEmOC hospitals because they cater for all or larger parts of the districts and therefore could not be randomized. For the women’s group trial, conditions (i) and (iii) were used as exclusion criteria, and urban facilities were also excluded because formative research indicated women’s groups are less likely to work in urban areas due to the transient nature of urban communities. Condition (ii) was also used as a de facto exclusion criteria for the community mobilization study as no women’s groups were set up in the areas surrounding dispensaries that do not carry out deliveries.

The average population of each health centre catchment area was approximately 30 000. We randomly sampled approximately 4000 people per cluster and set up a community surveillance system to track pregnancies, births and deaths of consenting women. Population mortality rates were derived from data collected by village development/health committee-approved volunteer key informants (KI), who captured all deaths.

Entry meetings with area development and district executive committees introduced the project to seek their approval. All pregnant women in surveillance areas who agreed to take part were eligible to be participants and enrolled if they became pregnant. In-migration of pregnant women to the open study cohort was allowed and occurred throughout the study period, as did outmigration.

### Description of interventions

#### Community mobilization

**Figure 2:**
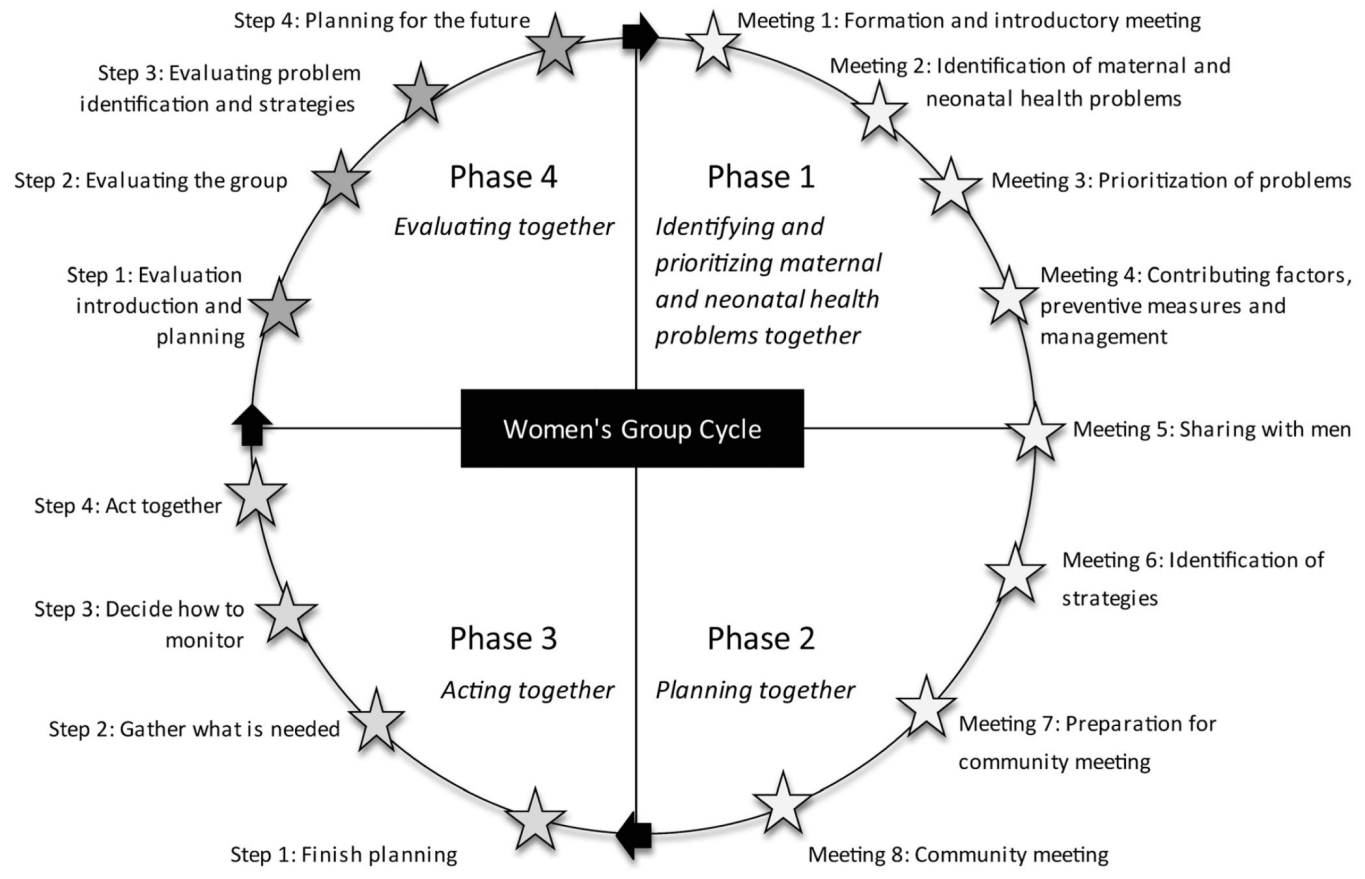
MaiKhanda Women’s Group Action Cycle followed by the women’s groups.

In mid-2007, MaiKhanda established 729 participatory women’s groups to mobilize communities around maternal and newborn health, using 81 volunteer facilitators, supported by nine staff, across the allocated clusters. The facilitators each formed nine village women’s groups which followed an ‘action cycle’, adapted from previous studies,^11^,^16^–^18^ to identify and prioritize maternal and neonatal health problems, decide upon local solutions, advocate for, implement and evaluate such strategies (Figure 2). The average women’s group size was 29 members in Kasungu, 37 in Lilongwe and 24 in Salima (phases I and II of the action cycle; Figure 2). Population coverage overall of one women’s group per 1200 population was slightly better than the BADAS trial in Bangladesh,^12^ (1:1400) but not as good as trials in India and Nepal.^10^ Only 10% of pregnancies in women’s group areas were among group members.

In mid-to-late 2009, 365 (50%) of the groups had maternal and neonatal health task forces (MNHTF) added to them by the MaiKhanda programme in an attempt to enhance antenatal coverage, maternal and neonatal health (MNH) knowledge, and facility delivery. MNHTF were set up by those women’s groups that chose MNH knowledge as a strategy to focus on. The MNHTF aimed to identify high-risk pregnant women; promote delivery at a health facility, and antenatal and postnatal care; and provide health education.

#### Quality improvement

**Figure 3:**
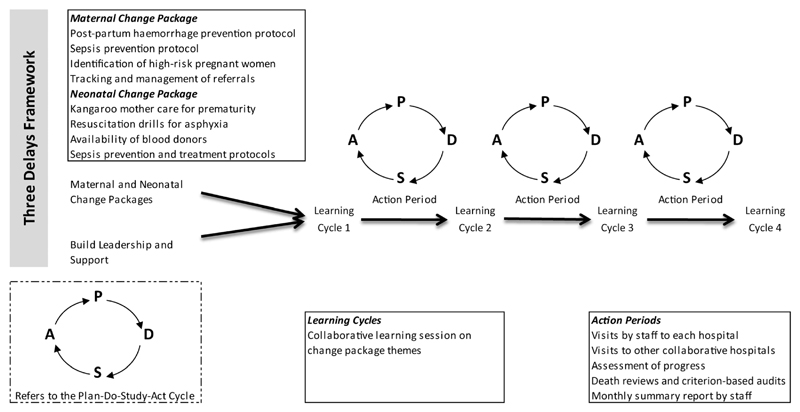
Overview of the facilities quality improvement intervention.

The quality improvement method consisted of breakthrough series collaboratives,^14^ and coaching of facility staff in quality improvement methodology, such as developing change ideas, conducting small tests of change using Plan-Do-Study-Act cycles, to improve care at health centres (within the RCT) and hospitals (outside the RCT). It also involved implementing change packages,^19^ and conducting death reviews^20^ and specific additional training, for local improvement leaders, and in situ training on specific clinical areas such as neonatal resuscitation drills and use of protocols for prevention and management of postpartum haemorrhage, sepsis and eclampsia. Figure 3 provides an overview of the different components.

The programme initially lacked sufficient staff to facilitate the full quality improvement intervention working with government staff as intended, and it was revised in mid-2008. Between 2006 and September 2009, quality improvement specialists on the MaiKhanda team grew from one to six. This delay meant that the FI was not fully implemented at the ‘dose’ anticipated from the study design. The programme targeted, with quality improvement, a limited number of the causes of maternal death (postpartum haemorrhage and sepsis via prevention protocols at hospitals; and referrals, identification of high-risk women and blood donor identification at the randomized health centres). The main causes of neonatal death were targeted for improvement (prematurity via kangaroo mother care, asphyxia via resuscitation drills, and sepsis via prevention and treatment protocols), but only from late-2009/early 2010.^9^

### Trial outline

**Figure 4:**
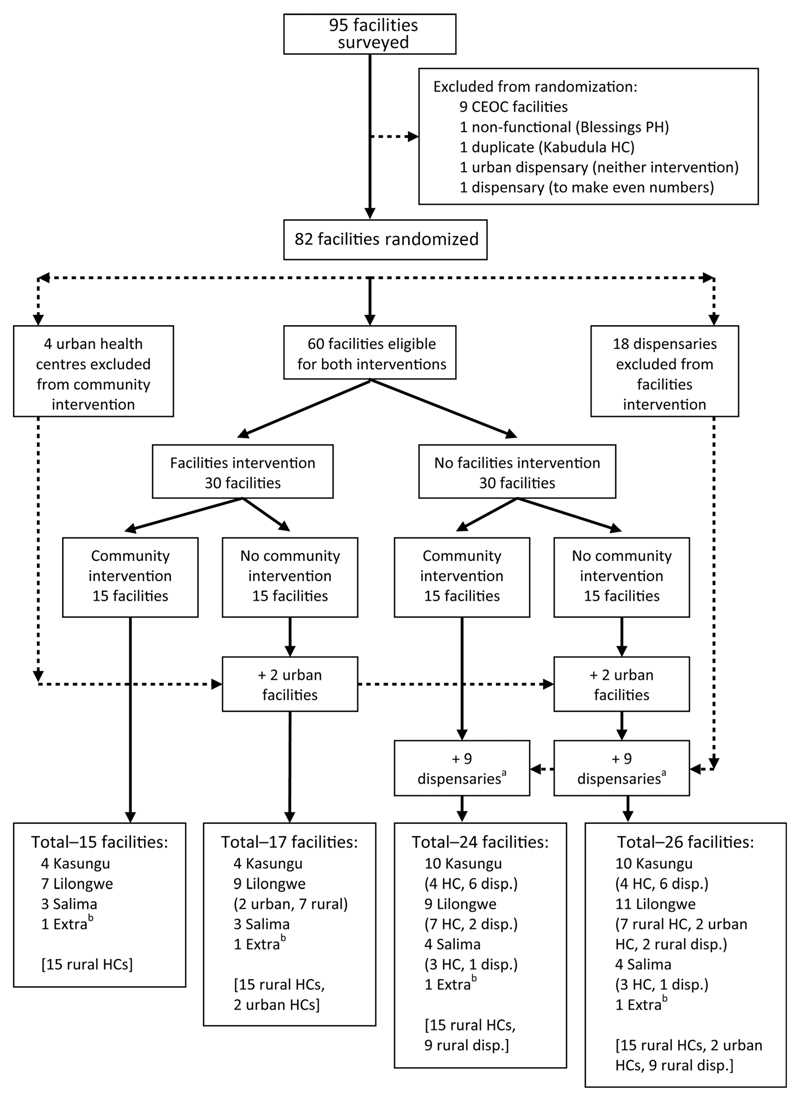
Randomization design flowchart. CEOC: comprehensive emergency obstetric care; disp.: dispensary; HC: health centre; PH: private hospital. ^a^Some of the rural clusters with dispensaries (HCs that do not do deliveries) that, according to the original trial design, were supposed to have women’s groups mistakenly never had women’s groups formed in them so there are actually 31 control clusters out of the 82 originally randomized to receive quality improvement and establishment of womens’ groups. ^b^The ‘extra’ facilities are three from Lilongwe and one from Kasungu that were left over after randomly allocating the others to each of the four intervention groups. To ensure the balance across interventions was maintained, they were allocated to each of the four intervention groups.

**Figure 5:**
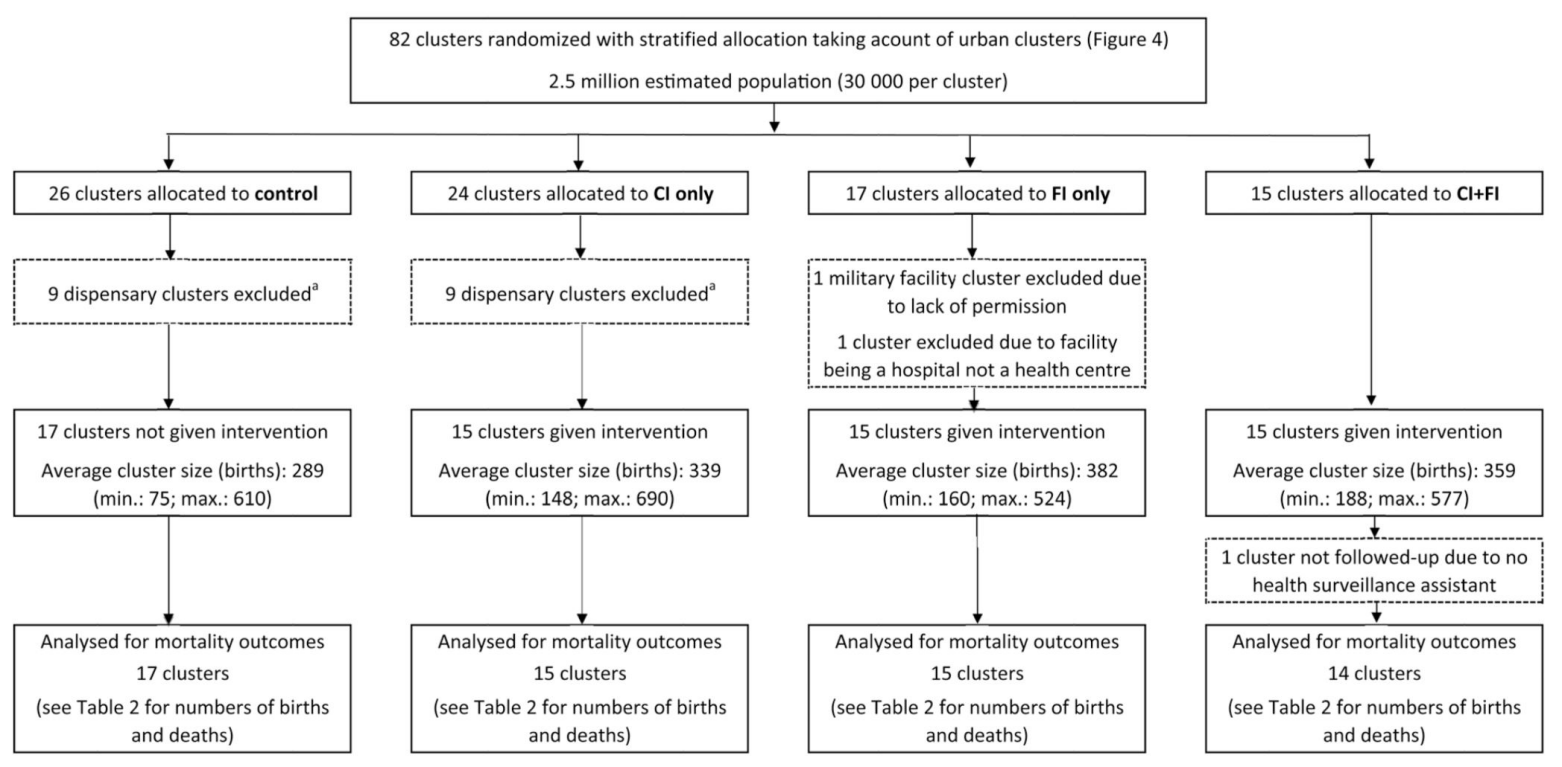
Trial profile flowchart (further details are provided in the technical report^9^). CI: community intervention; FI: facilities intervention. ^a^Dispensaries do not perform deliveries and were excluded as women’s groups were not set up in their catchment areas.

The interventions were tested by two trials combined in a factorial design producing four different groupings of intervention combinations (Figure 4). When MaiKhanda learned that the dispensary clusters did not do any deliveries, they were excluded from the trial before the interventions started, and were excluded from an intention-to-treat analysis as the intention was only ever briefly stated at the initial randomization, and removed after baseline information on delivery rates at health facilities was collected. After exclusion of two more FI clusters, one a military facility we did not get permission to work with and the other a district hospital rather than a health centre, there were 15 clusters with both interventions (CI + FI), 15 clusters with the FI only, 15 clusters with the CI only and 17 clusters with no interventions (control) (Figure 5). One CI + FI cluster could not have data collected in it due to a lack of community health workers, meaning it was excluded from the analysis, which involved 61 clusters in total.

There was no prespecified start date for the intervention. The intervention periods for both trials ran for 27 months from 1 October 2008 to 31 December 2010; with a preceding period of 16 months of baseline data collection: 1 June 2007 to 30 September 2008. The rationale for the intervention period beginning in October 2008 for the CI was the completion of the first two phases of the action cycle (Figure 2) by almost all women’s groups by September 2008; and the rationale for the quality improvement intervention was the completion of the first 90-day action cycle by the end of September 2008 following the relaunch of the intervention in the randomized health centres in July 2008.

### Randomization

Following the study design (Figure 4) clusters were allocated to each, both or no intervention with a random number sequence generated in Stata 7 (StataCorp LP, College Station, TX, USA). Randomization was stratified by the two interventions and by district, so that the numbers of intervention and control clusters in each district were balanced. To ensure concealment of intervention allocation, identification numbers were assigned for each cluster and a random number generated for each. The random numbers were sorted in ascending order, and a new ‘order’ variable generated. This sequence was used to allocate to each of the four intervention groups in each district. The sequence was concealed until interventions were assigned. SLgenerated the allocation sequence and assigned clusters to their groups in the presence of AC and MR. Given the nature of the interventions, neither participants nor those administering the interventions were blinded to group assignment. Those assessing the outcomes were also not blinded to group assignment. However, the analysis plan was prespecified and detailed in a Stata .do file before the final analysis was carried out.

### Primary and secondary outcomes

The primary outcome measures for both interventions were maternal, neonatal and perinatal mortality (Supplementary Box 1), assessed by monthly community surveillance of all pregnant women and their infants until 2 months after delivery throughout the 27-month intervention period. Any deaths of mothers or infants were followed-up with a verbal autopsy interview to verify and establish the cause of death.

Secondary outcome measures were: for both interventions: percentage of deliveries at a health facility; at facility level (for the quality improvement trial): percentage of maternal deaths subjected to maternal death audit, case–fatality rates, practice of signal obstetric care; at community level (for the women’s group trial): number of women’s groups mobilized annually (see below), percentage of pregnant women attending women’s groups. These were determined through monthly community surveillance, monthly health facility surveillance and collection of process data on the interventions.

### Community surveillance of births and deaths

Within each cluster (Figure 4) a population of approximately 4000 was randomly chosen as follows. We enumerated the size of the catchment populations of health surveillance assistants (HSA; government community health workers; initial average HSA catchment area size was a population of 2000) and then randomly chose HSAs from each cluster until a population of roughly 4000 was gained using the Complex Samples procedure in SPSS 14 (SPSS Inc., Chicago, IL, USA). Some cluster populations were significantly less or more than the desired 4000 population due to the large size of some HSA areas.

Village volunteers, called KIs, collected data for and were supervised by the HSAs. They recorded monthly data on pregnancies, miscarriages, abortions, stillbirths, live births, neonatal deaths, maternal deaths and place of delivery using a standard form. The form was translated into Chichewa, the main local language.

KIs were selected by local chiefs and MaiKhanda staff: criteria included literacy, basic numeracy, living locally and having the trust of the community. They went house to house to elicit the status of any pregnant women/new mothers every month, and covered about 50 households, or one village, typically following around five women each month. They were given T-shirts, and small allowances at quarterly refresher trainings. HSAs collected their forms each month and passed them on to MaiKhanda supervisors. Each HSA supervised around 5–10 KIs, and were paid small allowances at monthly meetings, and used the data collected by the KI for their own reports. HSAs were involved in surveillance only and not in either intervention.

Overall about 1800 KIs, worked in around 1900 villages throughout the three districts (the precise numbers changed throughout the surveillance as new hamlets were formed). Surveillance covered 8% of the rural population of Lilongwe district (around 150 000), 15% of Kasungu (90 000) and 21% of Salima (70 000).

### Data quality control

To ensure good quality data there was comprehensive training and monthly refresher meetings for KIs and HSAs by 11 monitoring and evaluation officers (MEOs) who were supervised by two evaluation investigators (BN and TC). Where data was unclear, callbacks to the woman or community were made. Unclear data on deaths were prioritized. Two trained data clerks entered data into a Microsoft Access database with validation rules. Duplicate records of women and newborn infants were identified and removed from the database, and the assignment of all women and all deaths in the database to the correct cluster and district was checked and amended as necessary. Deaths were verified by verbal autopsy interview with relatives of the mother or infant who died. A simple algorithm based on movement, breathing and crying of the baby was used to differentiate between stillbirths and neonatal deaths (see appendix 4 in the project technical report^9^).

### Statistical analysis

Sample size was determined using Hayes’ method with intercluster coefficient of variation.^21^ The final sample size was limited by the number of health facilities as the unit of randomization. The hypotheses being tested by the RCT were that each intervention would reduce maternal mortality by 30% (i.e. from 984 deaths per 100 000 at baseline to 688 deaths per 100 000) and neonatal mortality by 30% (i.e. from 27 to 19 deaths per 1000). Baseline data showed that the maternal mortality ratio was 415 instead of 984 deaths per 100 000 live births, so the power of the RCT to detect a 30% reduction was reduced to 36% (rather than the recommended 80% as originally designed). The RCT had an 80% power to detect reductions in maternal mortality ratio of 50% or larger. The power to detect a 30% reduction in neonatal mortality rate remained good at 98%: baseline neonatal mortality rate was 30.7/1000. The observed intracluster correlation coefficients in control clusters during the intervention period were 0.00118 for maternal and 0.00213 for neonatal, differing slightly to those reported earlier using preliminary data.^22^

### RCT analysis plan

Analyses were conducted in Stata 11.2 for Mac and planned in advance. After cleaning, data quality was assessed using cluster summaries of the percentages of women with no outcome data (loss to follow-up), maternal deaths with no verification, and stillbirths and neonatal deaths with no verification.

Descriptive analyses of time trends, annotated by baseline and intervention periods, for each of the primary outcome measures, by RCT arm and by district, assessed intervention effects and also initial balancing of the RCT arms with respect to baseline mortality rates.

Cluster-level analyses were made of intervention effects on mortality by weighted *t* test of cluster mortality rate summaries, taking stratification by district into account.^21^ Comparisons were made with and without the other intervention, and with and without adjustment for baseline mortality rates and other potential confounders such as health facility delivery, nurse/delivery ratio at health centre, signal function availability at health centre, baseline staff psychology score at health centre, urban/periurban/rural setting, access to health centre by tar road, health centre being either government-run or run by the Christian Health Association of Malawi, and clusters being tobacco estate areas (lots of migration) or not. Data for these confounders were collected during the baseline period (1 June 2007 to 30 September 2008), except for the nurse delivery ratio, collected by survey in 2010. The data, other than mortality, were collected from the health centres.^9^ The effect of each variable on mortality rates was assessed by descriptive univariate analyses followed by a stepwise logistic regression analysis of all variables.^23^

The cluster-level analyses were repeated at individual level using logistic regression with random effects by cluster.^21^ The results of these models are the primary results reported in this paper. Given that no data on individual level covariates was collected, adjustments were only made for cluster-level covariates. We adjusted for multiple hypothesis testing by adjusting reported p values of the primary outcomes using the Holm correction, which, when using a 5% significance level, adjusts the p value of each p < 0.05 result according to how many p < 0.05 results there are.^24^

We conducted the following secondary and exploratory analyses: analysis of the secondary outcome measures; assessment of trends in intervention effects (given changes to the dosage and content of the interventions) by splitting the intervention period into two: October 2008 to September 2009 and October 2009 to December 2010, the latter period being when the dosage of the FI was increased and when the task forces were added to the CI.

The data monitoring committee did not foresee any adverse effects of the interventions, so we did not apply stopping rules, interim analyses were not planned because they would have reduced power, and safety issues were not considered a problem.

### Additional analyses

Causes of perinatal and neonatal deaths were determined using the InterVA method.^25^ Process evaluation studies on health worker motivation and psychology, clinical knowledge of health workers, availability of human and material resources, the running of women’s groups using volunteers, and the success of the interventions with respect to their dosage, fidelity of implementation and sustainability, and contextual factors are reported elsewhere.^9^ Analysis of quality improvement efforts at nine hospitals, not in the RCT is in the project technical report.^9^ A detailed economic evaluation was conducted. Here we present headline results of the preliminary cost-effectiveness analysis.

## Results

After consideration of the CONSORT guidelines,^26^ deviations from protocol, and approval by the external data monitoring committee, changes were made to the number of clusters included in the final analysis and the trial profile is shown in Figure 5.

During the baseline period, the recorded loss to follow-up to birth outcomes was 19% and during the intervention it was 29%, with higher rates in later months. Given that observed birth rates in the study matched those expected from the crude birth rate^1^,^27^ to within 3%, and that in-migration probably broadly matched out-migration, many of the pregnancies recorded by KIs as ‘lost to follow-up’ may have been recorded as pregnancies by mistake and true loss-to-follow-up was probably much lower; there was little difference in loss-to-follow-up between arms.^9^ All 102 maternal deaths were verified—either through initial verbal autopsy (51) or by callbacks to the family later (51). A total of 300/2088 (14.4%) stillbirths and neonatal deaths remained unverified, despite a large catchup activity, with most women untraceable. The percentage unverified did not differ significantly by RCT arm. Despite verbal autopsy, some stillbirths could not be categorized correctly as macerated or fresh, nor neonatal deaths as early or late, necessary for calculation of perinatal and neonatal mortality rates (see Supplementary Box 1 definitions). There were no significant differences between the percentages of uncategorized stillbirths and neonatal deaths by RCT arm.

Table 1 shows potential confounding cluster-level variables at baseline for which we have data. Observed differences in baseline mortality, skilled birth attendance, urban, access by tar road, type of facility and tobacco estates were small and the variables were included in adjusted models if associated with the mortality outcome in question. Results of adjusted models were only slightly different to unadjusted models,^9^ and are not reported here.

When unadjusted for clustering or the factorial nature of the trial, there appeared to be a decline in stillbirths and perinatal deaths in the CI areas, and combined CI and FI area, relative to the other areas (Table 2). Splitting the intervention period into two, with the latter period representing increased dosage in the quality improvement, and increased time for effect and addition of task forces in the women’s groups (Supplementary Table 1) showed changes in mortality rates over time.

We observed a significant 22% reduction in neonatal mortality in the combined intervention clusters compared with the control arm (Table 3; OR = 0.78, 95% CI 0.60–1.01; p = 0.057). The CI and FI in combination led to a reduction from 34 to 27 neonatal deaths per 1000. We also observed a 16% reduction in perinatal mortality (OR = 0.84, 95% CI 0.72–0.97), in women’s group areas, adjusting for clustering, stratification and the presence of the quality improvement intervention (Table 3;p = 0.020), a reduction from 56 to 48 perinatal deaths per 1000. Our data were consistent with a 33% reduction in the relatively low rate of late neonatal deaths (OR = 0.67, 95% CI 0.46–0.97; p = 0.035; Table 3) in the facility improvement arm. We did not observe any significant reduction in neonatal or maternal mortality in women’s group areas. There was also no reduction in maternal, perinatal or neonatal mortality in quality improvement intervention areas. The results of the adjusted logistic regression analyses were similar but weaker to those of the unadjusted analyses and are presented in the technical report.^9^

**Figure 6:**
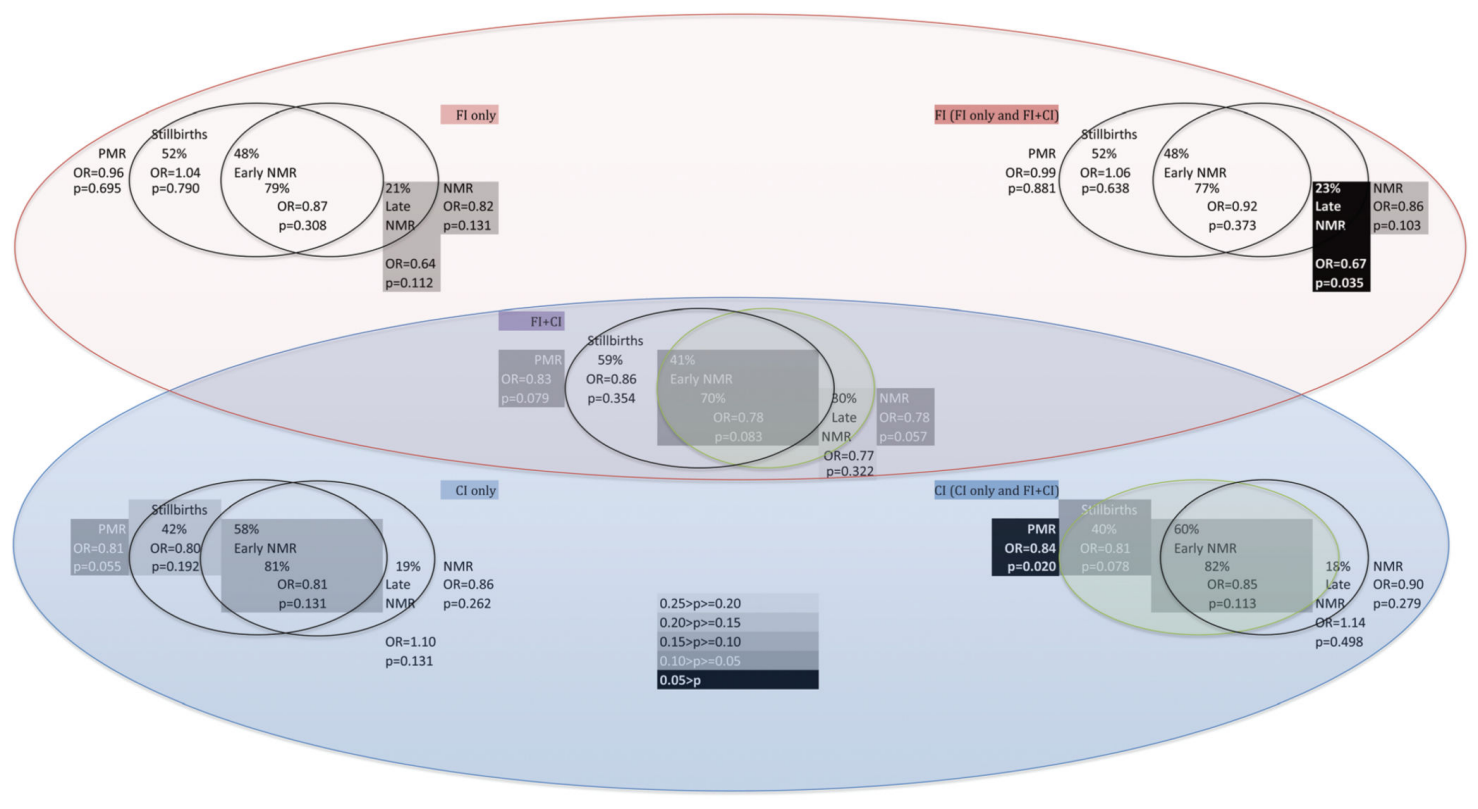
Internal consistency of randomized controlled trial (RCT) results. Percentages of observed PMR and NMR reductions due to reductions in stillbirth rate, early NMR, and late NMR by RCT comparison groups. CI: community intervention; FIL: facilities intervention; NMR: neonatal mortality rate; PMR: perinatal mortality rate.

Splitting the intervention period in two (to reflect the greater intensity in the second half of project implementation), and considering the cause of death data, suggests the observed effect of the combined FI and CI on neonatal mortality may have been mediated by reductions in mortality due to asphyxia, prematurity and sepsis, in both the early and late neonatal periods. The effect was mainly observed in the last, more intensive, 15 months of the intervention period, when it was larger (a 28% reduction; p = 0.084; Table 4); and, the effect of the CI on perinatal mortality may have been mediated through a reduction of stillbirths during the first 12 months of the intervention period and through a reduction in early neonatal mortality due to sepsis, prematurity and asphyxia in the second period of the study (Table 4). The internal consistency of the effects of both interventions on stillbirths and early and late neonatal deaths (the components of perinatal and neonatal mortality) is depicted in Figure 6. Increasing stillbirths and perinatal mortality in all areas, including the control area (Supplementary Table 1), is indicative of a secular trend rather than to do with dosage of the interventions. It is not clear why mortality rose at this time.

Given the observed number of births and deaths in each arm of the randomized trial the probability (calculated using the properties of the binomial distribution) that a 30% reduction in maternal mortality really occurred as a result of the MaiKhanda interventions in the total population our sample sought to represent (intervention areas vs control areas) was 1% for the FI and 28% for the CI.

Secondary outcome measures are reported in Table 5. While it was hypothesized that the CI would positively impact on both the decision and activity of women seeking skilled delivery, women’s groups had no effect on increasing health facility deliveries over and above the rapid increase observed in all areas (Table 5; Supplementary Figure 1) in line with the increase observed between the 2004 DHS survey (57%)^1^ and the 2010 DHS (73%).^2^ There were very few maternal deaths at health centres, which made comparison of FI intervention effects difficult. Neonatal case–fatality rates were low and not different between FI and control facilities. Fresh stillbirth rates were also low and were lower in FI facilities than control facilities in the intervention period. Although essential support systems were identified as one of the ‘key drivers’ of improved care, the availability of obstetric care signal functions were equally low across all comparison groups (Table 5), reflecting the fact that the health system is seriously under-resourced in Malawi.^28^,^29^

The proportion of newly pregnant women who attended women’s groups was 10% and the percentage of members who had never had children before was also low at 2%; we estimate a population coverage of approximately 1 women’s group to 1200 population.^9^ Given the results of recently published studies^10^,^12^ low coverage may help to explain a lower impact than Asian studies.

The effect of the CI on perinatal mortality and the effect of the FI and CI combined on neonatal mortality are highly cost- effective by WHO criteria (less than the per capita GDP of Malawi per disability-adjusted life-year averted, which is less than US$5400 per stillbirth or neonatal death averted).

## Discussion

In rural Malawi a combined facility improvement and women’s group community intervention reduced neonatal mortality by 22%, and a women’s group intervention alone reduced perinatal mortality by 16%, compared with control areas. No other study has measured the impact of supply and demand side interventions in Africa on maternal and newborn care, and population mortality outcomes, using a trial design.

Our results also suggest that the women’s groups had a greater effect on perinatal and early neonatal mortality, and the facility improvement on late neonatal mortality. The effects on mortality were greater in the latter stages of the effectiveness trial (Table 4), when the ‘dosage’ and implementation improved. Most newborn mortality reduction (due to the higher proportion of early neonatal deaths) arose probably from community mobilization, but was enhanced by efforts to improve quality at facilities. Larger effects of both interventions might be expected with more concentrated implementation.

Our study had limitations: use of a simple, large-scale surveillance system using KIs and government health workers may have led to some errors in stillbirth and neonatal death categorization. Without individual level covariates we were only able to undertake limited adjusted analyses using cluster-level covariates. After initial randomization we excluded several clusters when baseline information indicated that no deliveries took place at the health facilities (dispensaries) there. This exclusion was made before the start of the trial so we consider this does not break the intention to treat principle. The CEmOC hospital facilities excluded from the RCT were also undergoing quality improvement and women from both intervention and control areas delivered at these facilities. However, data from verbal autopsies show that only 19% of neonatal deaths and 51% of maternal deaths took place in these facilities rather than in health centres (17% neonatal deaths; 13% maternal deaths) or communities (56% neonatal deaths; 34% maternal deaths) and that these percentages were similar across all four RCT arms. The intervention effects we have measured are obviously independent of happenings at the CEmOCs. The verbal autopsy data also shows that <4% of deaths took place in other arms of the RCT from where the birth took place, meaning that ‘contamination’ between RCT arms is likely to have been minimal. ‘Spillover’ of intervention effects is also likely to have been minimal: for the CI because the influence of the women’s groups were contained within groups of villages under the same group village headmen, which are typically always within the same health facility catchment area (study cluster). In addition, opportunities for health centre clinicians to share quality improvement knowledge to clinicians in control health centres were very limited. The quality improvement and women’s group process evaluation was limited by the scale and resources of the intervention programme and the availability of government health services, in particular systems available for collection of routine health delivery data. Behaviour change interventions take time to work, so it is possible that stronger effects on service quality improvement and community mobilization will emerge with time. Quality improvement at district level in Malawi takes time to establish, and requires staff stability, leadership, training and ongoing support from district staff trained in quality improvement methods. The programme faced difficulties with each of these components in taking the programme to scale. Likewise, considerable effort, resources and commitment from the MoH and supporting NGOs are required to reach the concentration of women’s groups per population (1:500–750) to produce optimal improvements in neonatal mortality. This study achieved a lower coverage of 1:1200.

Our process evaluation showed a wide range of strategies implemented by women’s groups, with the most frequently implemented strategies being health education, voluntary testing and counselling for HIV/AIDS, village savings and loans, bednets, vegetable gardens and bicycle ambulances. Any of these strategies may have contributed to reductions in stillbirths and early neonatal deaths.^9^ For example, health education directed at antenatal, delivery and postnatal care could have improved corresponding practices, bicycle ambulances could have reduced delays in getting to health facilities, and village savings and loans could have enabled faster transport and provision of resources for mothers and infants. The strategies are proxy measures for communities having built their capacities to take control of mother and child health issues. This is an important mechanism through which women’s groups work—they help communities to take control of and address the social determinants of health (through empowerment) as well as the behavioural determinants (the strategies and behaviour changes).

The quality improvement intervention, in enhancing newborn mortality reduction from the combined intervention, appeared to have most effect on late neonatal deaths (Table 3). There was no clear evidence for the impact of specific quality improvement activities at the health centres on clinical practices such as identification of high-risk pregnant women and blood donors, or emergency obstetric referrals, although the linkage of improved clinical processes and outcomes may have been missed due to limited record keeping.^9^ Our data on signal functions at health centres shows no striking changes between the intervention and control health centres. Although fresh stillbirth rates were lower in quality improvement health centres, there was no effect of the intervention on neonatal case–fatality rates.

The lack of effect of either intervention on maternal mortality may reflect underdosing. The rapid increase in health facility delivery in Malawi, after the policy change regarding community births (Supplementary Figure 1), may have overwhelmed the poorly resourced health facilities, countering any benefits of the two interventions on women’s health. The potential for quality improvement to affect maternal deaths at the health facilities was limited because case–fatality rates at the health centres were very low and most deaths occurred at locations that were outside these centres—either in the community or at the referral hospitals. The combined CI and FI intervention together, but not alone, produced an effect on neonatal mortality rate, perhaps explained by weak independent effects which, when applied together achieved significance (Figure 6). Independent effects might have been detected if the interventions were fully dosed.

We know of no other randomized trials testing the effects of facility quality improvement interventions, or both facility and community interventions combined, on neonatal (or perinatal or maternal) mortality. Bhutta and colleagues^30^ concluded that strategies enhancing the skills of community health worker cadres demonstrated impact on stillbirths especially in combination with health systems strengthening activities. The Warmi project in Bolivia^16^ developed community mobilization through women’s groups. Subsequently, randomized trials to test the impact of women’s groups on neonatal mortality in Nepal,^11^ Jharkhand and Orissa in India^10^ and Bangladesh^12^ respectively, showed reductions of 30%, 45% and no significant fall in neonatal mortality. Effect sizes vary depending upon baseline mortality rates and levels of coverage by women’s groups, especially of newly pregnant women, and also contextual factors. Other studies, Hala in Pakistan^31^ and Shivgarh in India,^32^ which combined community mobilization and community health worker interventions showed neonatal mortality reductions of 15% and 54% respectively, although the latter was a small trial terminated after only 15 months.

A large review of quality improvement projects in poor settings showed better compliance with healthcare standards and improvement in health processes or outcomes for maternal and neonatal health, malaria, TB and HIV/AIDS.^14^ However, none of the outcomes were mortality rates; also the studies were uncontrolled and the data self-reported.^14^ An earlier systematic review^13^ on the evidence for quality improvement collaboratives, identified 57 (from a total of 72) studies that were based on the breakthrough series collaboratives. Seven were controlled, and one a RCT evaluating the breakthrough series integrated with the chronic care model. This trial did not show an effect on processes or intermediate outcomes of care for children with asthma.^33^ An update of the review found 10 controlled studies. Of these, two were RCTs: one showed no effect and the other showed an effect on two out of three processes but no effect on outcomes. Of the eight non-randomized, controlled studies, seven showed a positive effect on some of the selected effect parameters and one no effect.^34^ The USAID funded Health Care Improvement (HCI) project for maternal and newborn care in Niger^35^ looked mainly at performance measures but did not report on primary outcomes. Quality improvement based on performance and standards of care has recently also been evaluated in Malawi in a before and after study, but showed limited results and did not report on mortality outcomes.^36^

Ovretveit and Staines speak about an ‘investment threshold’ before quality improvement efforts start showing results.^37^ Initial investments in quality improvement do not necessarily show positive results but are important for institutionalization in the Malawian health care setting. The Zambian Quality Assurance Program established in 1993 covered the entire nation with a network of quality assurance coaches and trainers but the capacity to sustain quality assurance activities remained a challenge.^38^ It is unclear whether quality improvement approaches are more sustainable than quality assurance approaches in low-income settings. The Niger healthcare improvement project also had limited success in institutionalizing the quality improvement packages at scale for essential obstetric and neonatal care.^35^

Deming, who pioneered the use of Plan-Do-Study-Act cycles in quality improvement in the 1950s, emphasized the importance of long term commitment of management and leadership to transformation, and that those expecting quick results would be disappointed.^39^ Our study suggests that reducing facility case–fatality requires staff stability, leadership, training, ongoing support from district staff trained in quality improvement methods and external coaches who should make frequent visits.

We saw no trends in differences in population-level maternal mortality (Table 3), and no significant changes in maternal case–fatality rates (MCFR) between intervention and control health centres (Table 5), nor any significant impact on case–fatality of quality improvement work during 4 years at the nine non-randomized hospitals (median MCFRs were 381 deaths per 100 000 cases during January 2007 to December 2008, 266 during January 2009 to May 2010 and 310 during June 2010 to November 2011).^9^ Available process data on timing, dosage and focus, and on changes in causes of maternal death do not support a clear effect of the quality improvement on case–fatality at the nine CEmOCs.^9^ The observed early decrease in CEmOC MCFR was partially reversed in the second half of the period under review, possibly due to the overwhelming of resource-limited facilities by the surge in mothers seeking skilled deliveries over this period (due to a 2007 government ban on deliveries by traditional birth attendants).^9^ Probably, greater attention is needed on the ‘golden hour’ and ‘silver 6 hours’ of resuscitation and stabilization of severely sick mothers when they arrive at hospitals or health centres to make inroads into case–fatality. The quality improvement work in the nine CEmOCs did not have significant effects on neonatal case–fatality rates.^9^

Current Ministry of Health policy supports quality improvement and community mobilization interventions in principle,^40^–^42^ but resource allocation remains a major problem. Malawi has a double burden of HIV/AIDS and a human resource crisis.^43^ Our evaluation showed that health centres were severely under-resourced for the seven BEmOC signal functions and for human resources. Deficiencies of drugs and human resources were key factors impeding the essential health package across Malawi.^29^ Essential support systems were key drivers for change identified by the programme implementers.

Taking both interventions to scale at adequate coverage will need finance, integration with the Malawian Ministry of Health programme and technical assistance developed within country. There is a pressing need for further evaluation of how to achieve lasting impact at scale.

## Conclusions

Key conclusions for low-income countries like Malawi are that both communities and facilities should be targeted for improvement, and sufficient intensity must be delivered. The FI and CI in combination reduced neonatal mortality by 22% and had a greater effect (28% reduction, p = 0.084) in the later period (Table 4), when the intensity of the FI was greater, women’s groups were augmented by 365 task forces, and more time had elapsed. With major improvements in signal functions at facilities,^9^ the combined approach might reduce mortality rates more effectively, given rapidly rising deliveries in health centres and hospitals.

Both FI and CI improve motivation, solidarity, networks, and confidence in decision-making, which enable women and health workers to find solutions to problems. Both work to develop strategies and evaluate success using data. But there are also differences. CI focuses more on prevention (hygiene, infant feeding, social support) and the underlying causes of mortality (social isolation, poor nutrition, dangerous traditional practices, delays in seeking care). FI focuses more on improving the reliability of treatment of immediate causes, such as better management of haemorrhage or shock or asphyxia; and the data demands for women’s groups are less rigorous than for FI.

An integrated community and facility approach may be the best approach to reduce mortality. CI can hold facilities to account, and improved service quality enhances care-seeking. We cannot assume that quality improvement techniques, efficacious in a developed country setting, will transfer easily to resource-poor settings, so further rigorous evaluation is needed. However, as social and system interventions^44^ which improve birth outcomes and are highly cost-effective by WHO criteria,^9^ both deserve consideration for national and international policy.

## Supplementary data

Supplementary data are available at *International Health* Online (http://inthealth.oxfordjournals.org/).

Supplementary Data

Supplementary Figure 1

Supplementary Table 1

## Figures and Tables

**Table 1:** Baseline cluster-level characteristics by randomized controlled trial arm

Variable^a^		No FI	FI	No CI	CI	Control	FI	CI	FI + CI	Range^b^

No. of clusters		29	32	29	32	17	15	15	14	14 – 17
Baseline MMR	Mean	487	316	460	350	561	336	403	298	336–561
	SEM	107	73	94	95	147	104	160	105
Baseline NMR	Mean	30.7	28.5	32.5	26.7	31.8	33.3	29.4	24.0	24.0–33.3
	SEM	2.5	2.9	2.91	2.36	4.2	4.1	2.6	3.9	
Baseline PMR	Mean	57.0	54.6	55.9	55.8	55.5	56.4	58.7	52.9	52.9–58.7
	SEM	4.6	3.6	3.9	4.5	5.9	5.2	7.5	5.1	
Baseline skilled birth attendance (%)	Mean	45.5	50.1	48.8	46.5	49.6	47.9	40.8	52.2	40.8–52.2
	SEM	3.1	3.6	3.6	3.1	4.8	5.6	3.5	4.8	
Deliveries per month per nurse at nearest HC	Mean	21.2	25.2	22.9	23.4	21.4	24.8	21.0	25.6	21.4–25.6
	SEM	2.6	2.6	2.1	3.0	3.0	3.1	4.5	4.2	
Signal function availability at HC (no. of functions)	Mean	1.66	1.81	1.85	1.59	1.72	2.04	1.58	1.59	1.58–2.04
	SEM	0.24	0.28	0.2	0.3	0.25	0.35	0.43	0.43	
Baseline staff psychology score	Mean	2.40	2.46	2.31	2.53	2.29	2.35	2.52	2.54	2.29–2.54
(average of 19 questions on a scale of 1 [=bad] to 5 [=good])	SEM	0.07	0.07	0.08	0.06	0.09	0.16	0.10	0.06	
Urban (=1), periurban (=0.5) or rural (=0) setting	Mean	0.16	0.19	0.23	0.12	0.18	0.29	0.13	0.10	0.10–0.29
	SEM	0.06	0.06	0.07	0.05	0.1	0.11	0.08	0.05	
Access by tar road (=1) or dirt road (=0)	Mean	0.41	0.45	0.39	0.47	0.35	0.43	0.47	0.47	0.35–0.47
	SEM	0.09	0.09	0.09	0.09	0.12	0.14	0.13	0.13	
Christian Hospital Association of Malawi (=1) or government (=0) run	Mean	0.22	0.14	0.19	0.17	0.18	0.21	0.27	0.07	0.07–0.27
	SEM	0.07	0.07	0.07	0.07	0.1	0.11	0.12	0.07	
Tobacco estates (=1) or not (=0)	Mean	0.16	0.21	0.16	0.20	0.18	0.14	0.13	0.27	0.13–0.27
	SEM	0.07	0.08	0.07	0.07	0.1	0.10	0.09	0.12

Mean of cluster rates or ratios. Table 2 contains the overall rates or ratios for each study arm or group of two arms.

CI: community intervention; FI: facilities intervention; HC: health centre; MMR: maternal mortality ratio; NMR: neonatal mortality rate; PMR: perinatal mortality rate.

a No other data on characteristics of women, e.g. tribe, age, socioeconomic status or parity, was collected due to the simple and low-cost nature of the community surveillance system.

b Differences are not tested for statistical significance as, given the randomization process, the null hypothesis of no difference is already true, and any significant differences that were found would be due to chance.

**Table 2:** Baseline and intervention period mortality rates by randomized controlled trial arm, unadjusted for clustering or the factorial nature of the trial

	Baseline (1 June 2007 – 30 September 2008)								Intervention (1 October 2008 – 31 December 2010)							
	No FI	FI	No CI	CI	Control	FI only	CI only	FI + CI	No FI	FI	No CI	CI	Control	FI only	CI only	FI + CI

No.																
Births	7351	7225	7587	6989	3718	3869	3633	3356	9992	10584	10247	10329	4912	5335	5080	5249
Live births	7114	6993	7368	6739	3613	3755	3501	3238	9714	10272	9931	10055	4766	5165	4948	5107
Stillbirths	237	232	219	250	105	114	132	118	278	312	316	274	146	170	132	142
Mascerated	44	42	39	47	20	19	24	23	58	49	59	48	33	26	25	23
Fresh	128	110	120	118	59	61	69	49	127	165	162	130	67	95	60	70
Neonatal deaths	217	200	232	185	111	121	106	79	310	284	308	286	162	146	148	138
Early (0 – 6 days)	180	156	189	147	94	95	86	61	242	236	254	224	130	124	112	112
Late (7 – 28 days)	37	44	43	38	17	26	20	18	68	48	54	62	32	22	36	26
Perinatal deaths	417	388	408	397	199	209	218	179	520	548	570	498	276	294	244	254
Maternal deaths	29	26	29	26	15	14	14	12	21	26	25	22	10	15	11	11
Mortality rate																
Stillbirth rate per 1000 births	32.2	32.1	28.9	35.8	28.2	29.5	36.3	35.2	27.8	29.5	30.8	26.5	29.7	31.9	26.0	27.1
NMR per 1000 live births	30.5	28.6	31.5	27.5	30.7	32.2	30.3	24.4	31.9	27.6	31.0	28.4	34.0	28.3	29.9	27.0
Early NMR per 1000 live births (0 – 6 days)	25.3	22.3	25.7	21.8	26.0	25.3	24.6	18.8	24.9	23.0	25.6	22.3	27.3	24.0	22.6	21.9
Late NMR per 1000 live births (7 – 28 days)	5.2	6.3	5.8	5.6	4.7	6.9	5.7	5.6	7.0	4.7	5.4	6.2	6.7	4.3	7.3	5.1
PMR per 1000 births (stillbirths and early neonatal deaths)	56.7	53.7	53.8	56.8	53.5	54.0	60.0	53.3	52.0	51.8	55.6	48.2	56.2	55.1	48.0	48.4
MMR per 100 000 live births	407.6	371.8	393.6	385.8	415.2	372.8	399.9	370.6	216.2	253.1	251.7	218.8	209.8	290.4	222.3	215.4

CI: community intervention; FI: facilities intervention; MMR: maternal mortality ratio; NMR: neonatal mortality rate; PMR: perinatal mortality rate.

**Table 3:** Comparison of mortality rates in intervention and control clusters during the whole intervention period (1 October 2008 to 31 December 2010)

Outcome	2 × 2 factorial trial^a^				4 arm comparison^b^	
	FI vs no FI		CI vs no CI		FI + CI vs control	
	OR (95% CI)	p value	OR (95% CI)	p value	OR (95% CI)	p value

Stillbirth rate per 1000 births^c^	1.06 (0.84–1.32)	0.638	0.81 (0.65–1.02)	0.078	0.86 (0.62–1.19)	0.354
NMR per 1000 live births^d^	0.86 (0.72–1.03)	0.103	0.90 (0.75–1.09)	0.279	0.78 (0.60–1.01)	0.057
Early NMR per 1000 live births (0–6 days)^c^	0.92 (0.75–1.11)	0.373	0.85 (0.70–1.04)	0.113	0.78 (0.60–1.03)	0.083
Late NMR per 1000 livebirths (7–28 days)^c^	0.67 (0.46–0.97)	0.035	1.14 (0.78–1.66)	0.498	0.77 (0.45–1.30)	0.322
PMR per 1000 births (all stillbirths)^d^	0.99 (0.85–1.15)	0.881	0.84 (0.72–0.97)	0.020	0.83 (0.67–1.02)	0.079
MMR per 100 000 live births^d^	1.18 (0.66–2.11)	0.570	0.91 (0.51–1.63)	0.754	1.08 (0.46–2.57)	0.854

CI: community intervention; FI: facilities intervention; MMR: maternal mortality ratio; NMR: neonatal mortality rate; PMR: perinatal mortality rate.

a CI × FI interaction term not included as study underpowered to detect interactions.

b FI only vs control and CI only vs control results not shown.

c Secondary outcome.

d Primary outcome.

**Table 4:** Comparison of mortality rates in intervention and control clusters during the first and second intervention periods

	1st intervention period (1 October 2008 – 30 September 2009)						2nd intervention period (1 October 2009 – 31 December 2010)					
	2 × 2 factorial trial^a^				4 arm comparison^b^		2 × 2 factorial trial^a^				4 arm comparison^b^	
	FI vs no FI		CI vs no CI		FI + CI vs control		FI vs no FI		CI vs no CI		FI + CI vs control	
	OR (95% CI)	p value	OR (95% CI)	p value	OR (95% CI)	p value	OR (95% CI)	p value	OR (95% CI)	p value	OR (95% CI)	p value

Stillbirth rate per 1000 births^c^	1.18 (0.83–1.69)	0.360	0.70 (0.49–1.00)	0.051	0.82 (0.50–1.36)	0.454	0.97 (0.77–1.21)	0.767	0.94 (0.75–1.18)	0.621	0.91 (0.66–1.25)	0.576
NMR per 1000 live births^d^	0.82 (0.63–1.06)	0.124	1.05 (0.81–1.36)	0.700	0.86 (0.60–1.24)	0.423	0.90 (0.69–1.17)	0.431	0.80 (0.61–1.04)	0.098	0.72 (0.50–1.05)	0.084
Early NMR per 1000 live births (0–6 days)^c^	0.92 (0.67–1.26)	0.599	0.95 (0.69–1.29)	0.727	0.87 (0.56–1.35)	0.534	0.92 (0.70–1.19)	0.516	0.79 (0.60–1.03)	0.083	0.72 (0.49–1.06)	0.093
Late NMR per 1000 live births (7–28 days)^c^	0.54 (0.31–0.92)	0.025	1.62 (0.93–2.80)	0.089	0.87 (0.39–1.91)	0.722	0.83 (0.49–1.39)	0.473	0.81 (0.48–1.37)	0.432	0.69 (0.34–1.40)	0.303
PMR per 1000 births (all stillbirths)^d^	1.04 (0.83–1.31)	0.719	0.81 (0.65–1.02)	0.074	0.85 (0.61–1.17)	0.309	0.94 (0.77–1.15)	0.555	0.87 (0.71–1.06)	0.179	0.82 (0.62–1.09)	0.171
MMR per 100 000 live births^d^	1.91 (0.82–4.48)	0.135	1.37 (0.60–3.09)	0.455	3.62 (0.77–17.07)	0.105	0.74 (0.32–1.69)	0.476	0.58 (0.24–1.39)	0.223	0.40 (0.11–1.54)	0.184

CI: community Intervention; FI: facilities intervention; MMR: maternal mortality ratio; NMR: neonatal mortality rate: PMR: perinatal mortality rate.

a CI × FI interaction term not included as study underpowered to detect interactions.

b FI only vs control and CI only vs control results not shown.

c Secondary outcome.

d Primary outcome.

**Table 5:** Secondary outcome measures by randomized controlled trial arm

Secondary outcome measure		Baseline (1 June 2007–30 September 2008)				Intervention (1 October 2008–31 December 2010)			
		Control	CI only	FI only	FI + CI	Control	CI only	FI only	FI + CI

Mean % of deliveries at a health facility	%	50	41	48	52	67	58	67	70
	SEM	5	4	6	5	4	4	4	4

		No FI		FI		No FI		FI	

Deliveries at health centres		18 286		20 234		43 326		44 172	
Maternal deaths at health centres	No.	7		10		12		8	
	Audited	5		5		1		0	
	MCFR [mean (95% CI)]	38 (19–79)		49 (27–91)		28 (16–48)		18 (9–36)	
Neonatal deaths at health centres	No.	95		99		219		201
	NCFR [mean (95% CI)]	5.2 (4.3–6.3)		4.9 (4.0–6.0)		5.1 (4.4–5.8)		4.6 (4–5.2)
Fresh stillbirths at health centres	No.	123		117		339		244	
	FSBR [mean (95% CI)]	6.7 (5.6–8.0)		5.8 (4.8–6.9)		7.8 (7.0–8.7)		5.5 (4.9–6.3)	
Availability of signal functions (%)^a^									
Manual removal of placenta		12		7		14		13	
Manual vacuum aspiration		5		1		5		8	
Vacuum extraction		2		15		5		18	
Breech deliveries		43		45		28		38	
Parenteral antibiotics		33		25		29		32	
Magnesium sulphate		15		22		14		20	
Oxytocic drugs (ergometrine or oxytocin)		54		44		39		48	
Caesarian section		0		0		0		0	
Blood transfusion		5		0		2		0	

CI: community intervention; FI: facilities intervention; FSBR: fresh stillbirth rate (fresh stillbirths per 1000 deliveries); MCFR: maternal case – fatality rate (maternal deaths per 100 000 deliveries); NCFR: neonatal case – fatality rate (neonatal deaths per 1000 deliveries).

a Percentage of months where signal functions were available at health centres (health centres equally weighted). The first 7 signal functions listed are basic emergency obstetric care (BEmOC) signal functions; the last two are comprehensive emergency obstetric care (CEmOC) signal functions.

## References

[1] National Statistical Office (NSO), ORC Macro (2005). Malawi Demographic and Health Survey 2004.

[2] National Statistical Office (NSO), ICF Macro (2011). Malawi Demographic and Health Survey 2010.

[3] Bhutta ZA, Chopra M, Axelson H (2010). Countdown to 2015 decade report (2000–2010): taking stock of maternal, newborn, and child survival. Lancet.

[4] Zimba E, Kinney MV, Kachale F (2012). Newborn survival in Malawi: a decade of change and future implications. Health Policy Plan.

[5] Republic of Malawi Ministry of Health (2010). Malawi 2010 EmONC Needs Assessment Final Report.

[6] Costello A, Filippi VA, Kubba T, Horton R (2007). Research challenges to improve maternal and child survival. Lancet.

[7] Thaddeus S, Maine D (1994). Too far to walk: maternal mortality in context. Soc Sci Med.

[8] Institute for Healthcare Improvement, Liverpool Associates in Tropical Health, Liverpool School of Tropical Medicine, UCL Institute of Child Health (2005). Proposal to The Health Foundation for Improvement of the Quality of Maternal and Neonatal Care in Malawi.

[9] Colbourn T, Nambiar B, Costello A (2013). MaiKhanda – Final Evaluation Report. The Impact of Quality Improvement at Health Facilities and Community Mobilisation by Women’s Groups on Birth Outcomes: an Effectiveness Study in Three Districts Of Malawi.

[10] Tripathy P, Nair N, Barnett S (2010). Effect of a participatory intervention with women’s groups on birth outcomes and maternal depression in Jharkhand and Orissa, India: a cluster-randomised controlled trial. Lancet.

[11] Manandhar DS, Osrin D, Shrestha BP (2004). Effect of a participatory intervention with women’s groups on birth outcomes in Nepal: cluster-randomised controlled trial. Lancet.

[12] Azad K, Barnett S, Banerjee B (2010). Effect of scaling up women’s groups on birth outcomes in three rural districts in Bangladesh: a cluster-randomised controlled trial. Lancet.

[13] Schouten LM, Hulscher ME, van Everdingen JJE (2008). Evidence for the impact of quality improvement collaboratives: a systematic review. BMJ.

[14] Franco LM, Marquez L (2011). Effectiveness of collaborative improvement: evidence from 27 applications in 12 less-developed and middle-income countries. BMJ Qual Saf.

[15] UNDP (2011). Human Development Report 2011. Sustainability and Equity: A Better Future for All.

[16] O’Rourke K, Howard-Grabman L, Seoane G (1998). Impact of community organization of women on perinatal outcomes in rural Bolivia. Rev Panam Salud Publica.

[17] Lewycka S, Mwansambo C, Kazembe P (2010). A cluster randomised controlled trial of the community effectiveness of two interventions in rural Malawi to improve health care and to reduce maternal, newborn and infant mortality. Trials.

[18] Rosato M, Mwansambo C, Lewycka S (2010). MaiMwana women’s groups: a community mobilisation intervention to improve mother and child health and reduce mortality in rural Malawi. Malawi Med J.

[19] Langley GJ, Moen R, Nolan KM (2009). The Improvement Guide: A Practical Approach to Enhancing Organizational Performance.

[20] Kongnyuy EJ, Mlava G, van den Broek N (2009). Facility-based maternal death review in three districts in the central region of Malawi an analysis of causes and characteristics of maternal deaths. Womens Health Issues.

[21] Hayes R, Moulton LH (2009). Cluster Randomised Controlled Trials.

[22] Pagel C, Prost A, Lewycka S (2011). Intracluster correlation coefficients and coefficients of variation for perinatal outcomes from five cluster-randomised controlled trials in low and middle-income countries: results and methodological implications. Trials.

[23] Hosmer DW, Lemeshow S (2000). Applied Logistic Regression. Wiley Series in Probability and Statistics.

[24] Holm S (1979). A simple sequentially rejective multiple test procedure. Scand J Statist.

[25] Vergnano S, Fottrell E, Osrin D (2011). Adaptation of a probabilistic method (InterVA) of verbal autopsy to improve the interpretation of cause of stillbirth and neonatal death in Malawi, Nepal, and Zimbabwe. Popul Health Metr.

[26] Campbell MK, Elbourne DR, Altman DG (2004). CONSORTstatement: extension to cluster randomised trials. Brit Med J.

[27] National Statistical Office (NSO), UNICEF (2008). Malawi Multiple Indicator Cluster Survey 2006, Final Report.

[28] Leigh B, Mwale TG, Lazaro D, Lunguzi J (2008). Emergency obstetric care: how do we stand in Malawi?. Int J Gynecol Obstet.

[29] Mueller DH, Lungu D, Acharya A, Palmer N (2011). Constraints to implementing the Essential Health Package in Malawi. PLoS One.

[30] Bhutta ZA, Darmstadt GL, Haws RA (2009). Delivering interventions to reduce the global burden of stillbirths: improving service supply and community demand. BMC Pregnancy Childbirth.

[31] Bhutta ZA, Soofi S, Cousens S (2011). Improvement of perinatal and newborn care in rural Pakistan through community-based strategies: a cluster-randomised effectiveness trial. Lancet.

[32] Kumar V, Mohanty S, Kumar A (2008). Effect of community-based behaviour change management on neonatal mortality in Shivgarh, Uttar Pradesh, India: a cluster-randomised controlled trial. Lancet.

[33] Homer CJ, Forbes P, Horvitz L (2005). Impact of a quality improvement program on care and outcomes for children with asthma. Arch Pediatr Adolesc Med.

[34] Hulscher M, Schouten L, Grol R, Collaboratives (2009). Quest for Quality and Improved Performance.

[35] Boucar M, Franco LM, Sabou D (2011). Sustaining Better Maternal and Newborn Care and Quality Improvement in Niger: Challenges and Successes. Research and Evaluation Report. USAID Health Care Improvement Project.

[36] Rawlins BJ, Kim Y-M, Rozario AM (2013). Reproductive health services in Malawi: an evaluation of a quality improvement intervention. Midwifery.

[37] Ovretveit J, Staines A (2007). Sustained improvement? Findings from an independent case study of the Jonkoping quality program. Qual Manag Health Care.

[38] Bouchet B, Francisco M, Ovretveit J (2002). The Zambia quality assurance program: successes and challenges. Int J Qual Health Care.

[39] Deming WE (1986). Out of the Crisis.

[40] Republic of Malawi Ministry of Health (2011). Road Map for Accelerating the Reduction of Maternal and Neonatal Morbidity and Mortality in Malawi.

[41] Government of Malawi Ministry of Health (2012). Malawi Health Sector Strategic Plan 2011-2016. Moving Towards Equity and Quality.

[42] Government of Malawi Ministry of Health GTZ Health Sector Programme (October 29th 2010) Quality Improvement of Health Care Services in Malawi. Objective, Strategies, and Key Interventions for Building Block 4 of the Health SWAp POW II.

[43] McCoy D, McPake B, Mwapasa V (2008). The double burden of human resource and HIV crises: a case study of Malawi. Hum Res Health.

[44] Osrin D, Prost A (2010). Perinatal interventions and survival in resource-poor settings: which work, which don’t, which have the jury out?. Arch Dis Child.

