# Supplementary material for: Effects of quality improvement in health facilities and community mobilization through women’s groups on maternal, neonatal and perinatal mortality in three districts of Malawi: MaiKhanda, a cluster randomized controlled effectiveness trial
Source: Int Health. Author manuscript; Available in PMC 2016 Nov 9. (PMC5102328; doi:10.1093/inthealth/iht011)
Supplement: Supplementary Data [file NIHMS70208-supplement-Supplementary_Data.docx]

**Supplementary Box 1. Mortality definitions**

**Stillbirth:** “A stillbirth or foetal death is a death prior to the complete expulsion or extraction from its mother of a product of conception, irrespective of the duration of pregnancy; the death is indicated by the fact that after such separation the foetus does not breathe or show any other evidence of life, such as beating of the heart, pulsation of the umbilical cord or definite movement of voluntary muscles.” We used the ICD-10 criteria for stillbirth modified to include births after 28 completed weeks rather than 22 weeks.

**Neonatal death:** death within the first 28 days of an infant after “the complete expulsion or extraction from its mother of a product of conception, irrespective of the duration of the pregnancy, which after such separation, breathes or shows any other evidence of life, such as beating of the heart, pulsation of the umbilical cord, or definite movement of voluntary muscles, whether or not the umbilical cord has been cut or the placenta is attached.”

**Perinatal death:** stillbirth or neonatal death within the first 7 days

**Maternal death:** “the death of a woman while pregnant or within 42 days**^a^** of termination of pregnancy, irrespective of the duration and site of the pregnancy, from any cause related to or aggravated by the pregnancy or its management but not from accidental or incidental causes.”

^a^We included three maternal deaths after 42 days: one at 50 days (CI only arm); one at 51 days (FI+CI arm) and one at 57 days (FI+CI arm) after birth. They all died of maternal causes, and although after 42 days after delivery, died within the 2-month follow-up period (maternal deaths after 60 days were excluded because we did not follow-up women past 60 days).
