# Supplementary material for: Effects of quality improvement in health facilities and community mobilization through women’s groups on maternal, neonatal and perinatal mortality in three districts of Malawi: MaiKhanda, a cluster randomized controlled effectiveness trial
Source: Int Health. Author manuscript; Available in PMC 2016 Nov 9. (PMC5102328; doi:10.1093/inthealth/iht011)
Supplement: Supplementary Figure 1 [file NIHMS70208-supplement-Supplementary_Figure_1.doc]

Supplementary Figure 1.
